# Perfluorooctanesulfonic acid modulates barrier function and systemic T-cell homeostasis during intestinal inflammation

**DOI:** 10.1242/dmm.049104

**Published:** 2021-12-23

**Authors:** Oscar E. Diaz, Chiara Sorini, Rodrigo A. Morales, Xinxin Luo, Annika Frede, Annette M. Krais, Myra N. Chávez, Emma Wincent, Srustidhar Das, Eduardo J. Villablanca

**Affiliations:** 1 Division of Immunology and Allergy, Department of Medicine, Solna, Karolinska Institutet and University Hospital, 17176 Stockholm, Sweden; 2 Center of Molecular Medicine, 17176 Stockholm, Sweden; 3 Division of Occupational and Environmental Medicine, Institution of Laboratory Medicine, Lund University, 22363 Lund, Sweden; 4 Institute of Anatomy, University of Bern, Baltzerstr. 2, 3012 Bern, Switzerland; 5 Institute of Environmental Medicine, Karolinska Institutet, Nobels väg 13, 171 77 Solna, Sweden

**Keywords:** T cell, Colitis, Experimental models, Inflammation, Pollutants

## Abstract

The intestinal epithelium is continuously exposed to deleterious environmental factors that might cause aberrant immune responses leading to inflammatory disorders. However, what environmental factors might contribute to disease are poorly understood. Here, to overcome the lack of *in vivo* models suitable for screening of environmental factors, we used zebrafish reporters of intestinal inflammation. Using zebrafish, we interrogated the immunomodulatory effects of polyfluoroalkyl substances, which have been positively associated with ulcerative colitis incidence. Exposure to perfluorooctanesulfonic acid (PFOS) during 2,4,6-trinitro-benzene sulfonic acid (TNBS)-induced inflammation enhanced the expression of proinflammatory cytokines as well as neutrophil recruitment to the intestine of zebrafish larvae, which was validated in the TNBS-induced colitis mouse model. Moreover, PFOS exposure in mice undergoing colitis resulted in neutrophil-dependent increased intestinal permeability and enhanced PFOS translocation into the circulation. This was associated with a neutrophil-dependent expansion of systemic CD4^+^ T cells. Thus, our results indicate that PFOS worsens inflammation-induced intestinal damage with disruption of T-cell homeostasis beyond the gut and provides a novel *in vivo* toolbox to screen for pollutants affecting intestinal homeostasis.

## Abstract (precis)

**Summary:** Oral exposure to a widely distributed pollutant resulted in exacerbated intestinal inflammation in zebrafish and mice, increased barrier permeability and altered T-cell homeostasis.

## INTRODUCTION

Inflammatory bowel diseases (IBDs), encompassing Crohn's disease and ulcerative colitis, are multifactorial diseases characterized by chronic inflammation of the gastrointestinal tract. The etiology of IBD has not been fully elucidated, but it is believed to develop as an active and continuing inflammatory response triggered by environmental factors in genetically susceptible hosts (Ananthakrishnan, 2015; Khor et al., 2011). The prevalence of IBD has historically been higher in Western countries, although it is now a global disease, with the highest increase in incidence in newly developing countries (Kaplan and Ng, 2017). This shift in IBD epidemiology reinforces the notion that environmental factors play an important role in disease pathogenesis (Ananthakrishnan et al., 2018). However, a major challenge in the field of mucosal immunology is the identification of environmental factors that might disrupt intestinal homeostasis and consequently lead to intestinal inflammation.

To fill these knowledge gaps, it is imperative to develop relevant and cost-effective experimental models to interrogate the effects of emerging environmental factors on intestinal physiology (Ho et al., 2019). Although cell-based high-throughput screening (HTS) approaches allow the interrogation of thousands of compounds and conditions, it remains challenging to translate findings into an *in vivo* context. More biologically relevant are *in vivo* settings, in which experimental murine models of colitis have provided critical knowledge in the field of mucosal immunology. However, owing to the large number of potential environmental factors, it is not feasible to use murine models. Zebrafish has emerged as an alternative experimental model to study mucosal immunology (Diaz et al., 2020; Jijon et al., 2018; Kaya et al., 2020; Ulhaq et al., 2013; Ye et al., 2021, 2019). Among the advantages of the zebrafish system are (1) their small size, allowing their growth in 96-well-plates; (2) their reduced cost of husbandry and high fecundity (∼200 embryos per cross); (3) their transparency, allowing non-invasive imaging; and (4) the possibility of exposures to small molecules by immersion without the need for injections. Thus, zebrafish embryos can be considered a versatile model system to investigate the effects of environmental factors on mucosal immunity.

Among environmental factors, exposure to dietary factors and pollutants represents a risk factor for developing IBD (Ananthakrishnan, 2015; Ho et al., 2019; Kish et al., 2013). Among emerging chemical pollutants, per- and polyfluoroalkyl substances (PFASs) are highlighted as high-risk chemicals causing both adverse health effects and substantial costs to society. PFASs are anthropogenic, highly persistent and mobile compounds with a global distribution. Owing to their chemical properties, including water repellency and high resistance to fire, they are used in a variety of products, including cookware, firefighting foams and water-repellent textiles (Blum et al., 2015; Herzke et al., 2012). Of specific concern is the increasing PFAS contamination of drinking water and ground water due to industrial waste pollution or firefighting activities, resulting in increased exposure to a high number of individuals. Notably, perfluorooctanesulfonic acid (PFOS) and perfluorooctanoic acid (PFOA), the most-studied members of this family of compounds, have shown adverse immune effects, such as reduced efficiency of vaccines and suppression of humoral immune responses, and have been positively associated with incidence of ulcerative colitis (Blum et al., 2015; Grandjean et al., 2012; Granum et al., 2013; Peden-Adams et al., 2008; Steenland et al., 2013). Moreover, PFOS exposure was shown to be detrimental in the clearance of an intestinal bacterial infection by modulating the response of innate lymphoid cells (Suo et al., 2017). In line with this, a recent report by the European Food Safety Authority (EFSA) identified effects on the immune system as the most critical for the risk assessment of PFASs [EFSA Panel on Contaminants in the Food Chain (EFSA CONTAM Panel), 2020]. Owing to health concerns associated with exposure to the longer-chain-length PFASs, i.e. PFOS and PFOA, these have been listed as Persistent Organic Pollutants in the Stockholm Convention and have been replaced with compounds of shorter chain length, such as perfluorohexanesulfonic acid (PFHxS), which are deemed less bioaccumulative. However, these compounds are still highly persistent in the environment, and a characterization of biological effects from their exposure is still lacking (Blum et al., 2015). Moreover, the mechanism through which this class of environmental pollutants modulates immune responses at early stages of intestinal inflammation and its effect on the intestinal barrier function remain largely unclear.

In this study, we have assessed the immunomodulatory effects of PFASs using a chemically induced zebrafish model of intestinal inflammation. Among the PFASs tested, we observed that PFOS exacerbates an ongoing inflammation, as seen by increased neutrophil recruitment to the gastrointestinal tract as well as higher expression of proinflammatory cytokines. To translate our findings to mammals, we exposed mice undergoing intestinal inflammation to PFOS, which resulted in enhanced neutrophil infiltration to the colon. Mechanistically, PFOS affects intestinal permeability in mice, which was associated with increased PFOS uptake and accumulation in systemic organs such as the liver. Oral administration of PFOS showed consequences in the adaptive immune system beyond the gut, as seen by a systemic expansion of CD4^+^ T cells such as in the spleen. Altogether, our results validated the use of zebrafish as a reliable model to identify environmental factors that might disrupt intestinal homeostasis and identified PFOS as a detrimental environmental factor that exacerbates intestinal barrier disruption in an ongoing inflammatory response.

## RESULTS

### Zebrafish as a model to study intestinal inflammation

**Fig. 1.**
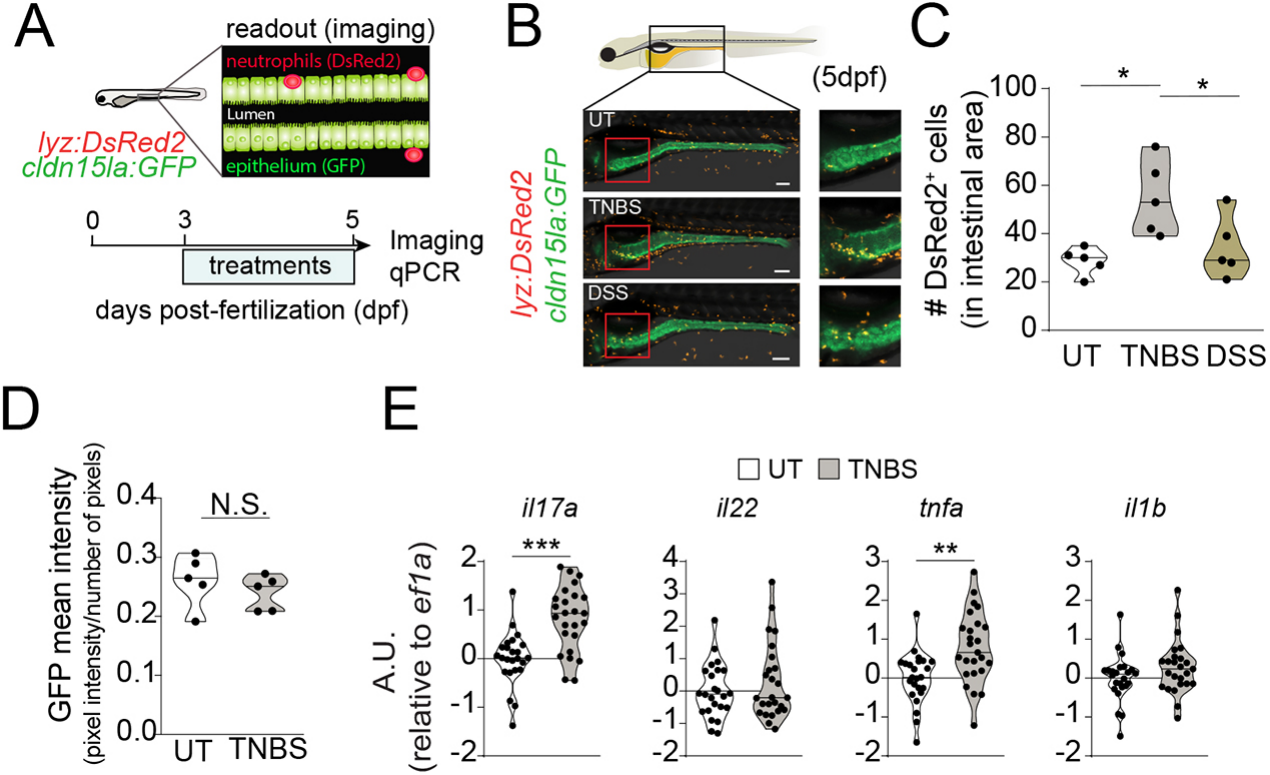
**2,4,6-trinitro-benzene sulfonic acid (TNBS) induces intestinal inflammation in zebrafish larvae.** (A) Experimental outline. *Tg(lyz:DsRed2, cldn15la:GFP)* zebrafish larvae were exposed to TNBS (50 µg/ml) or dextran sodium sulphate (DSS) (0.5%) from 72 hours post-fertilization (hpf) until 120 hpf. (B) Confocal microscopy images of DSS, TNBS and untreated (UT) *Tg(lyz:DsRed2, cldn15la:GFP)* larvae at 5× magnification at 120 hpf. Green fluorescence marks intestinal epithelial cells. Red fluorescence marks neutrophils. Scale bars: 100 µm. (C,D) Quantification of DsRed2^+^ cells in the intestine (C) and mean GFP^+^ intensity (sum of pixel intensities per number of pixels) (D). *n*=5, one experiment. Each data point represents one 120 hpf zebrafish larva. (E) Violin plots showing the relative expression of proinflammatory cytokines *il1b*, *tnfa*, *il17a/f3* and *il22* analyzed by qPCR in whole larvae at 120 hpf following exposure to TNBS (70 µg/ml). *n*=24-25, nine experiments. Data show transcript levels as arbitrary units (A.U.) with respect to *eef1a1l1* (indicated as *ef1a*). Each dot represents a pool of ten zebrafish larvae. The black line represents the median. N.S., not significant; *P<0.05, **P<0.01, ***P<0.001. One-way ANOVA with Fisher's LSD test was used in C; unpaired Student's *t*-test was used in D and E.

In order to establish an experimental zebrafish model of intestinal inflammation compatible with screening approaches, we tested distinct chemicals known to damage the intestinal epithelium and trigger inflammation in mouse (Antoniou et al., 2016). Therefore, we took advantage of the *TgBAC(cldn15la:GFP)* (intestinal epithelium specific, herein referred to as *cldn15la:GFP*) (Alvers et al., 2014) crossed to the *Tg(lysC:DsRed2)* (neutrophil specific, herein referred to as *lyz:DsRed2*) (Hall et al., 2007) to generate a double reporter zebrafish line that enables the visualization of neutrophil recruitment to the intestine, which is considered a hallmark of intestinal inflammation. We exposed the *Tg(lyz:DsRed2, cldn15la:GFP)* double reporter larvae to either 2,4,6-trinitro-benzene sulfonic acid (TNBS) or dextran sodium sulphate (DSS) (Oehlers et al., 2013, 2017) from 72 h post fertilization (hpf) to 120 hpf (Fig. 1A) and analyzed neutrophil recruitment to the intestine. We observed that TNBS, but not DSS, resulted in enhanced neutrophil recruitment to the intestine (Fig. 1B,C), suggesting that TNBS was able to induce intestinal inflammation. This was not accompanied by differences in GFP intensity between untreated and TNBS-treated larvae, suggesting no gross alterations in the intestinal epithelium (Fig. 1D). However, in line with increased neutrophil influx, we observed upregulation of *il17a* and *tnfa*, which are cytokines among others known to be induced during intestinal inflammation in mouse and humans (Czarnewski et al., 2019; Neurath, 2014) (Fig. 1E). Altogether, TNBS triggered an inflammatory response in zebrafish larvae that may be monitored in HTS approaches.

### PFOS enhances *il1b* expression in zebrafish larvae undergoing chemically induced intestinal inflammation

**Fig. 2.**
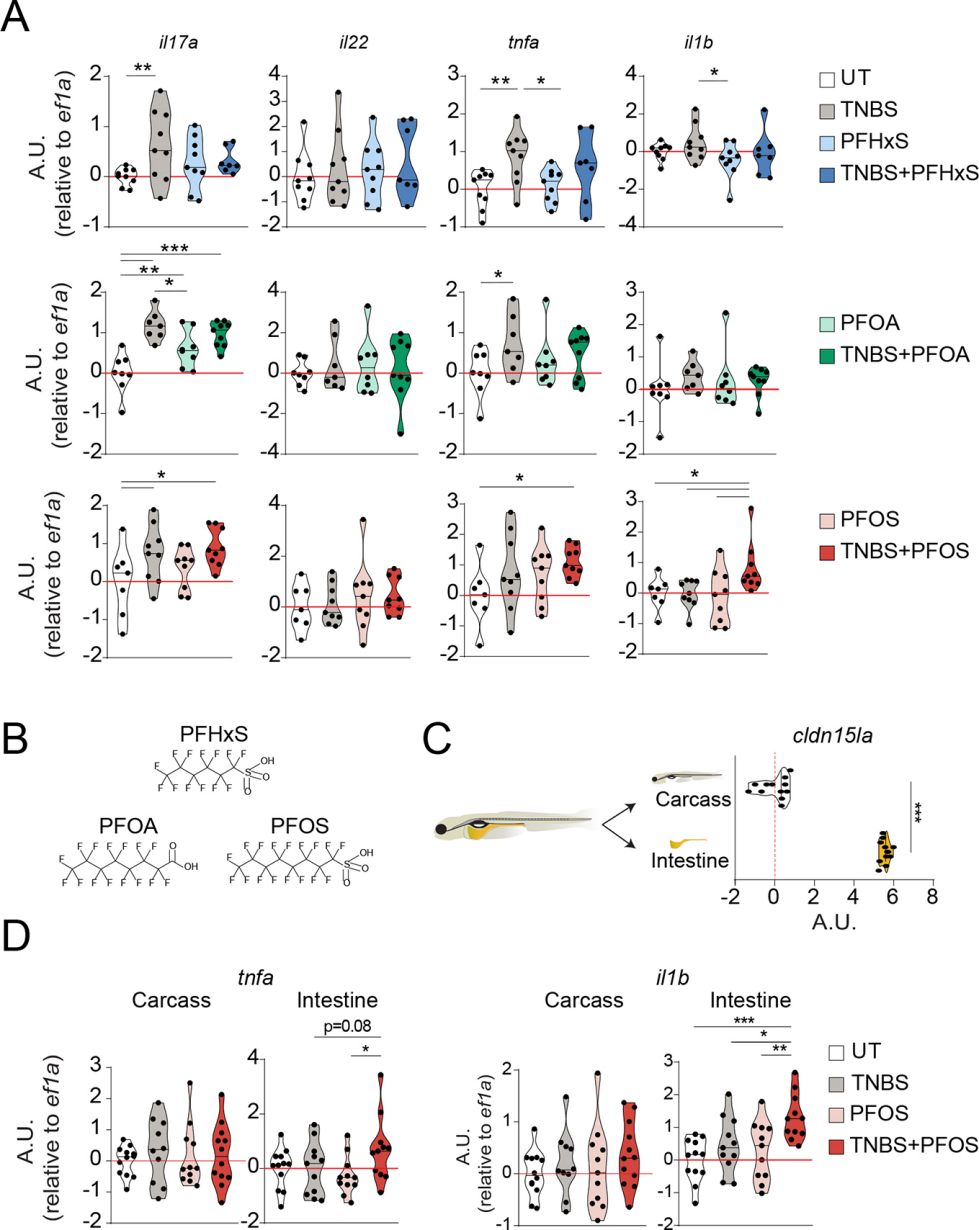
**Perfluorooctanesulfonic acid (PFOS) exacerbates intestinal-specific expression of proinflammatory cytokines during TNBS-induced inflammation in zebrafish larvae.** (A) Zebrafish larvae were exposed to TNBS (70 µg/ml) and/or PFOS, perfluorooctanoic acid (PFOA) or perfluorohexanesulfonic acid (PFHxS) (200 nM) from 72 hpf until 120 hpf. Violin plots showing the relative expression of *il1b*, *tnfa*, *il17a/f3* and *il22* in whole larvae at 120 hpf following exposure to TNBS and PFOS, PFOA or PFHxS. *n*=7-9, three experiments. Each dot represents a pool of ten zebrafish larvae. Values for the UT and TNBS groups for the experiment with each individual polyfluoroalkyl substance (PFAS) in A have previously been presented as a pool in Fig. 1D. (B) Panel of PFASs tested. (C,D) Scheme of dissection of intestines from zebrafish larvae (C, left) and violin plots showing relative expression of an intestine-specific gene, *cldn15la* (C, right), and proinflammatory cytokines, *tnfa* (D, left) and *il1b* (D, right), analyzed by qPCR in dissected intestines or carcasses at 120 hpf following exposure to TNBS (70 µg/ml) and PFOS (200 nM). *n*=11-12, four experiments. Data show transcript levels as A.U. with respect to *eef1l1a1*. Each dot represents a pool of ten intestines or carcasses. The black line represents the median. *P<0.05, **P<0.01, ***P<0.001. One-way ANOVA with Fisher's LSD test for all plots, except in C, for which an unpaired Student's *t*-test was used.

Next, we sought to investigate whether TNBS-treatment in zebrafish can be used to identify pollutants that might contribute to intestinal inflammation. We focused on PFASs, which have been associated with ulcerative colitis in humans exposed to increased environmental levels, through ingestion of contaminated water and diet (Blum et al., 2015; Grandjean et al., 2012; Granum et al., 2013; Peden-Adams et al., 2008; Steenland et al., 2013). To evaluate the effect of PFAS exposure on intestinal inflammation, we tested the effects of three widely environmentally spread PFASs (PFOS, PFOA and PFHxS) in zebrafish larvae exposed to TNBS (Oehlers et al., 2013) from 72 hpf to 120 hpf (Fig. 2A,B). The PFOS concentration (200 nM) used was based on the lowest observed effect level (LOEL) in zebrafish larvae at 120 hpf, which had been previously reported (Hagenaars et al., 2011; Peden-Adams et al., 2008). The same concentration was used for PFOA and PFHxS to have better comparability. Although exposure to any of the PFASs (e.g. PFOA) tested resulted in only slight increases in *il17a* expression in the absence of inflammation, exposure to PFOS, but not to PFOA or PFHxS, resulted in enhanced expression of *il17a*, *tnfa* and *il1b* in zebrafish larvae undergoing TNBS-induced inflammation (Fig. 2A). To determine whether the increased expression of these cytokines seen in whole larvae upon TNBS-PFOS co-exposure was intestine specific, we dissected larval intestines and validated the enrichment of intestinal tissues by analyzing the transcript levels of *cldn15la* (Alvers et al., 2014) (Fig. 2C). Despite changes in the carcasses, the intestinal expression of *il17a* and *il22* was not significantly affected by PFOS exposure during inflammation, compared to that in the TNBS group (Fig. S1A). Similarly, we observed that intestinal expression of immune cell markers for macrophages (*mpeg1.1*), neutrophils (*lyz*) and lymphocytes (*lck*, *trac*), as well as tight junction proteins (*tjap1*), was comparable between zebrafish exposed to TNBS in the presence or absence of PFOS (Fig. S1B). Further, histological evaluation using Hematoxylin and Eosin (H&E) did not show any abnormality in the intestinal tissue architecture upon co-exposure to TNBS and PFOS (Fig. S2A). Likewise, we did not detect differences in goblet cell numbers, as seen by Alcian Blue staining (Fig. S2B) between the TNBS+PFOS treatment and untreated groups. Overall, this suggests that the presence of PFOS during intestinal inflammation does not result in gross changes in intestinal morphology. Interestingly, we found that exposure to PFOS during inflammation slightly increased intestinal *tnfa* expression, albeit not significantly, while resulting in a significantly higher *il1b* expression in the intestine, compared to TNBS alone (Fig. 2D). This effect seems to be specific to the intestine because it triggered only a slight, but nonsignificant, increase in *il1b* expression in the carcasses. Altogether, these data suggest that PFOS exacerbates intestinal inflammation in zebrafish.

### PFOS enhances neutrophil recruitment during intestinal inflammation in zebrafish larvae and mice

**Fig. 3.**
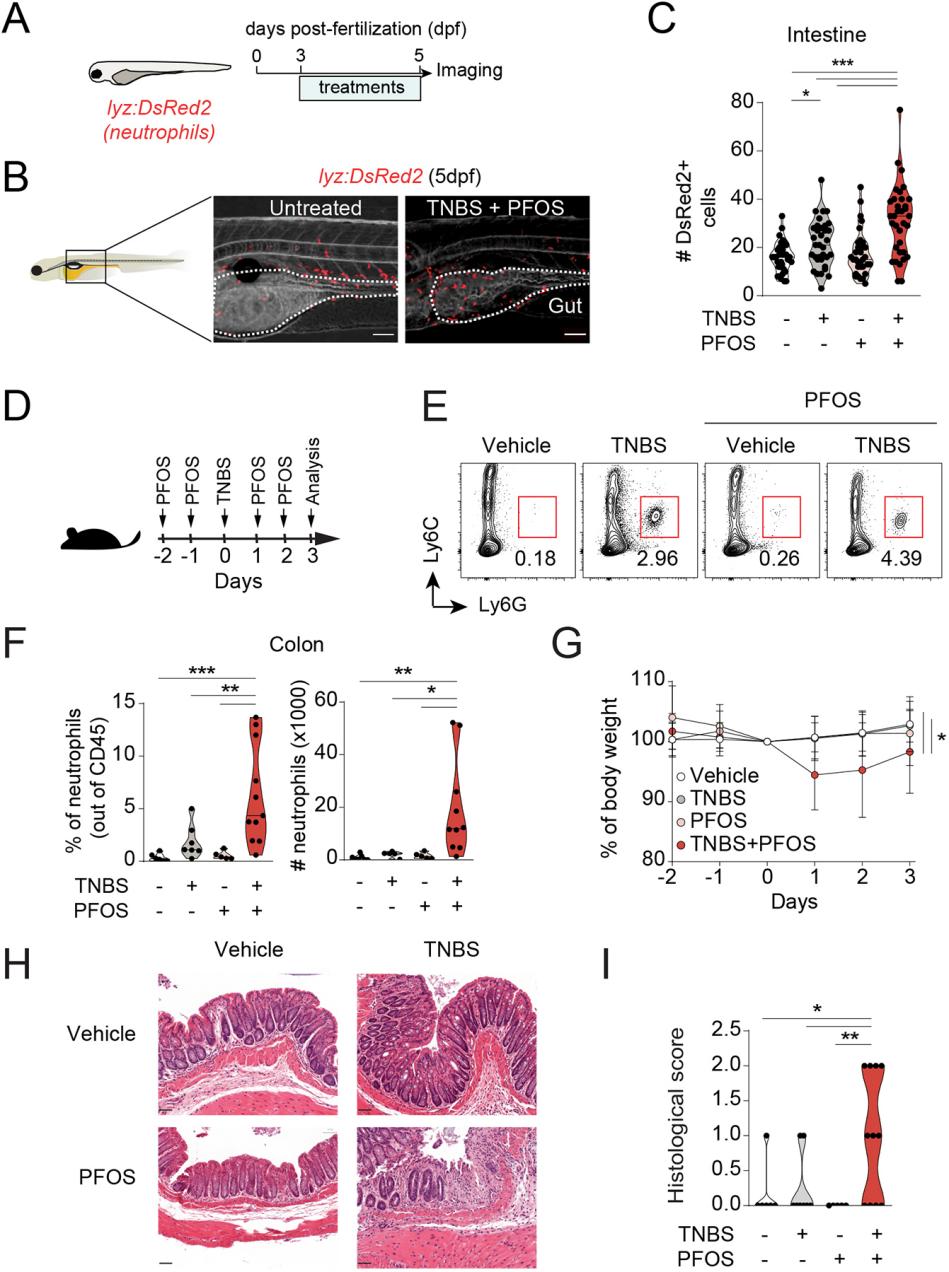
**PFOS increases neutrophil recruitment to the intestine during TNBS-induced intestinal inflammation in zebrafish larvae and mice.** (A) Experimental outline. *Tg(lyz:DsRed2)* zebrafish larvae were exposed to TNBS (50 µg/ml) and PFOS (200 nM) from 72 hpf until 120 hpf. (B) High-resolution light-sheet microscopy images of TNBS+PFOS and untreated *lysC:DsRed2* larvae at 5× magnification at 120 hpf. The intestine is marked in yellow and outlined in white. Red fluorescence marks neutrophils. Scale bars: 100 µm. (C) Quantification of DsRed2^+^ cells in the intestine. *n*=32-35, five experiments. Each data point represents one 120 hpf zebrafish larva. The black line represents the median. (D) Experimental outline. Mice received a single dose of TNBS (1%, in 50% ethanol, intrarectally administered) and four doses of PFOS (10 mg/kg/total dose, orally gavaged). (E) Flow cytometry analysis of neutrophils isolated from the colon lamina propria. CD45^+^ cells were gated out for analysis of Ly6C and Ly6G. Neutrophils are gated as Ly6C^int^ and Ly6G^+^. *n*=5-11, three experiments. (F) Violin plots showing neutrophil frequencies out of CD45^+^ cells and absolute numbers. (G) Body weight loss curves from mice treated with TNBS and PFOS. (H) Representative H&E microscopy images of distal colons from mice treated with TNBS and PFOS. Scale bars: 50 µm. (I) Violin plots showing histological scores quantified from H&E staining of colon sections as shown in H. *n*=5-11, three experiments. *P<0.05, **P<0.01, ***P<0.001. One-way ANOVA with Fisher's LSD test.

Neutrophil recruitment to the intestine, in particular in response to IL-1β, remains one of the major signs of intestinal inflammation in humans (Muthas et al., 2017) and mice (Fournier and Parkos, 2012). To address whether neutrophil recruitment to the gastrointestinal tract was altered by PFOS exposure in larvae undergoing TNBS-induced intestinal inflammation, we used the *lyz:DsRed2* reporter larvae. We observed that PFOS exposure exacerbated neutrophil recruitment specifically during intestinal inflammation (Fig. 3A-C). Moreover, this effect was not seen in larvae treated with PFOS alone. To investigate whether this effect was due to a systemic neutrophil expansion rather than recruitment, we analyzed the number of neutrophils in the aorta-gonad-mesonephros and caudal hematopoietic tissue (CHT), which are major sites of hematopoiesis at this developmental stage. Whereas TNBS induced an increase in neutrophils in CHT, this was not altered by PFOS co-exposure (Fig. S3A), indicating that neutrophils are not expanded systemically. Moreover, considering the mild increase, albeit not significant, in *mpeg1.1* expression in the intestine of PFOS-exposed larvae during inflammation, we wondered whether macrophage recruitment to the gastrointestinal tract was affected. To address this, we imaged *mpeg1:mCherry-F* zebrafish larvae (Nguyen-Chi et al., 2017) and found an increase in macrophage recruitment to the intestine of TNBS-treated larvae, which was not further increased by PFOS exposure (Fig. S3B).

To test the relevance of our findings from our zebrafish model of intestinal inflammation, we studied the effect of PFOS exposure in the corresponding murine model of colitis. To determine whether exposure to PFOS exacerbates intestinal inflammation, mice received a suboptimal intrarectal dose of TNBS and were orally gavaged with PFOS before and after induction of colitis. This would allow us to assess the effects of PFOS without inducing overt inflammation (Fig. 3D). The PFOS concentration used [10 mg/kg total administered dose (TAD)] was chosen based on the LOEL in a prolonged exposure (60-day period, 5 mg/kg TAD) (Dong et al., 2009) but below 35 mg/kg/TAD, which was reported to be well tolerated without triggering major changes in the immune cell compartment following a 7-day treatment (Zheng et al., 2009).

Consistent with our observations in zebrafish, we found increased frequencies and numbers of neutrophils in mice co-exposed to TNBS and PFOS compared to those exposed to TNBS alone (Fig. 3E,F). When analyzing other myeloid cells, we also found an increase in the number, but not frequencies, of monocytes, in colitic mice that were exposed to TNBS and PFOS compared to those only treated with TNBS (Fig. S4A-C). In agreement, TNBS only triggered a suboptimal inflammation without overt changes in body weight and minor changes in histological scores. On the other hand, mice co-exposed to TNBS and PFOS displayed significant loss of body weight, higher histological score and reduced colon length (Fig. 3G-I; Fig. S4D). Thus, PFOS exacerbates neutrophil recruitment to the intestine during TNBS-induced intestinal inflammation in both zebrafish and mice.

### PFOS exposure during intestinal inflammation results in increased intestinal permeability and translocation into the circulation

Because the principal route of PFOS intake in humans remains through contaminated water and diet (Haug et al., 2011), which can potentially affect the gut microbiota, we asked the question whether PFOS exposure during colitis might alter the composition of bacterial taxa in the gastrointestinal tract. Real-time quantitative PCR (qPCR) analysis showed comparable abundance of Gamma Proteobacteria, Firmicutes, *Lactobacillus* and segmented filamentous bacteria in mice undergoing intestinal inflammation exposed or not to PFOS (Fig. S5). We only detected a reduction in Bacteroide (mouse intestinal Bacteroides) abundance in TNBS-treated mice. Overall, this suggests that PFOS did not dramatically alter the gut microbiota composition during intestinal inflammation.

**Fig. 4.**
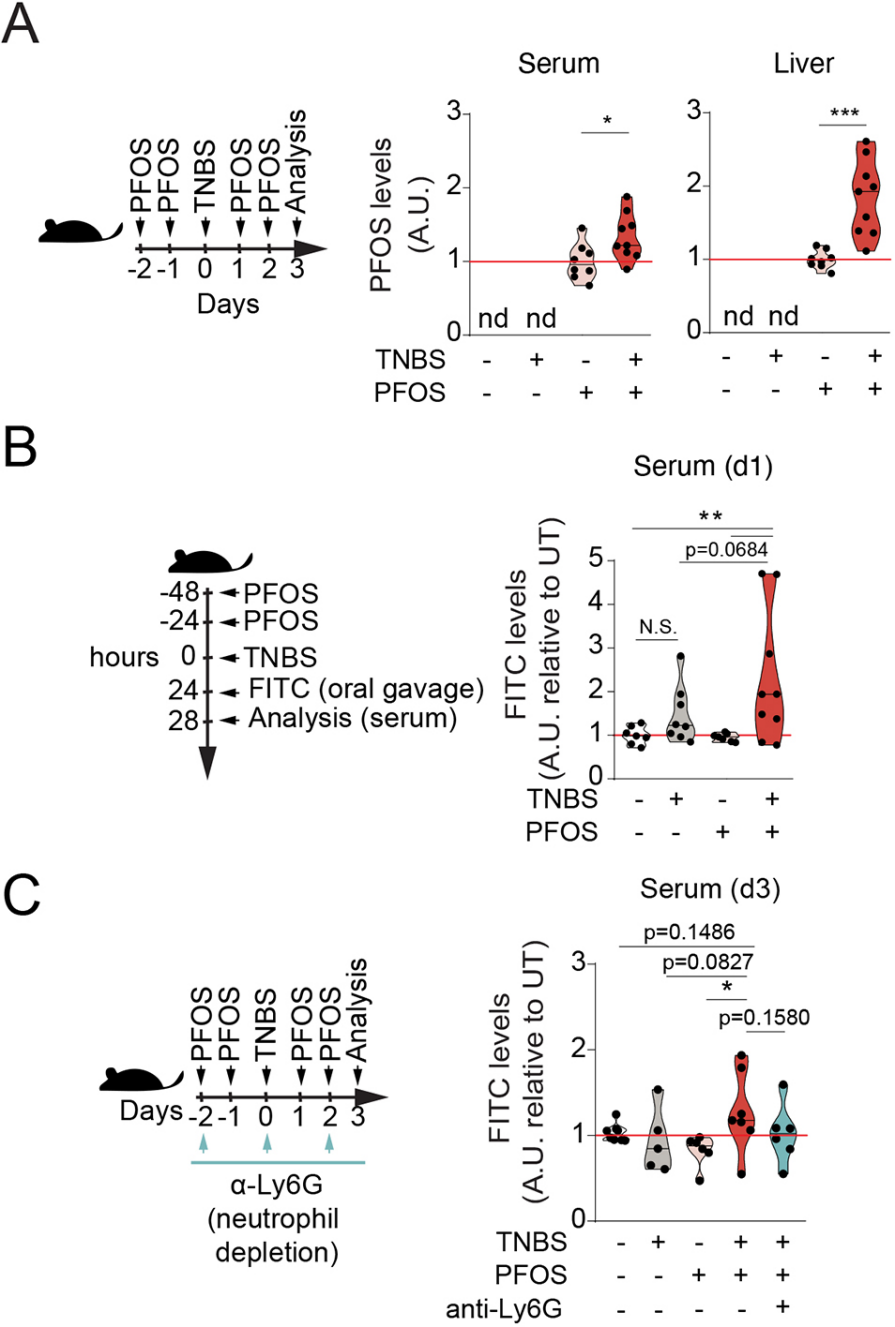
**PFOS exposure during colitis leads to increased systemic levels and intestinal permeability in mice.** (A) Violin plots represent normalized PFOS levels detected in serum (middle) and liver (right) at day 3 after TNBS administration, following the experimental outline shown in the scheme (left). Values have been calculated as fold change of the average concentration detected in the PFOS group of each experiment. Values for vehicle and TNBS groups were not detected (nd). *n*=8-9, three experiments. (B) Intestinal permeability to 4 kDa FITC-Dextran measured in serum at 4 h after oral gavage at day 1 following TNBS administration and PFOS (5 mg/kg/total dose), following the experimental outline shown in the scheme. Data shown in violin plots relative to the average of the values of the vehicle group in each experiment. *n*=7-9, three experiments. (C) Mice received a single dose of TNBS (1%, in 50% ethanol, intrarectally administered), four doses of PFOS (10 mg/kg/total dose, orally gavaged) and 500 µg per dose of anti-Ly6G monoclonal antibody every other day, following the experimental outline shown in the scheme. Intestinal permeability measured in serum at 4 h after oral gavage at day 3 post-TNBS administration. Data shown in violin plots are relative to the average of the values of the vehicle group in each experiment. *n*=5-7, three experiments. The black line represents the median. N.S., not significant; *P<0.05, **P<0.01, ***P<0.001. One-way ANOVA with Fisher's LSD test.

PFOS has been detected in the circulation following long-term exposures in mice (Peden-Adams et al., 2008), suggesting that orally administered PFOS might translocate into the blood. Indeed, we detected higher PFOS levels in the circulation as well as in the liver, an organ in which it is known to bioaccumulate, in mice undergoing TNBS-induced colitis at day 3 post-TNBS administration (Fig. 4A). We then asked whether this increased translocation and bioaccumulation of PFOS following a short exposure is a result of impaired epithelial permeability. Considering that increased intestinal permeability has been reported to be an early event that precedes intestinal inflammation in both mice and humans (Arrieta et al., 2009; Turpin et al., 2020), we first performed a fluorescein isothiocyanate (FITC)-Dextran permeability assay within 24 h of colitis induction (Fig. S6A,B). We observed that, although PFOS alone did not increase intestinal permeability, exposure to PFOS during intestinal inflammation resulted in increased FITC levels detected in serum (Fig. 4B), indicating increased permeability. Of note, regardless of PFOS exposure, there was marked neutrophil recruitment to the colon of TNBS-treated mice at this time point (Fig. S6B,C). Interestingly, on day 3, whereas the neutrophil numbers in TNBS-treated mice almost returned to baseline, mice co-exposed to TNBS-PFOS still showed significantly higher neutrophil numbers in the colon compared to the TNBS-treated mice (Fig. 3E,F), suggesting that PFOS exposure results in persistent neutrophil recruitment during intestinal inflammation.

Next, to test whether the impaired barrier function was sustained upon PFOS exposure, we analyzed intestinal barrier permeability at day 3 after colitis induction. Although the impaired permeability returned to baseline upon TNBS treatment compared to controls on day 3, when co-exposed with PFOS, a trend (*P*=0.1486, although not significant) in the impaired permeability was still observed (Fig. 4C).

Moreover, to test whether the increased neutrophils had any bearing on intestinal permeability, we took advantage of the anti-Ly6G neutrophil-depleting antibody (Daley et al., 2008). Following administration of anti-Ly6G antibody prior to and during TNBS-induced colitis and PFOS exposure, we observed decreased serum levels of FITC-Dextran, which were close to the levels detected in the group exposed to vehicle and PFOS alone, thus suggesting that the increased permeability seen upon TNBS+PFOS co-exposure was, at least partially, due to the increased neutrophil influx to the colon in TNBS and PFOS co-exposed mice (Fig. 4C; Fig. S6D). Together, these results indicated that PFOS exposure during TNBS-induced colitis results in impaired intestinal barrier function, partly mediated by neutrophil influx.

### PFOS exposure during intestinal inflammation results in peripheral CD4^+^ T-cell expansion that is neutrophil dependent

**Fig. 5.**
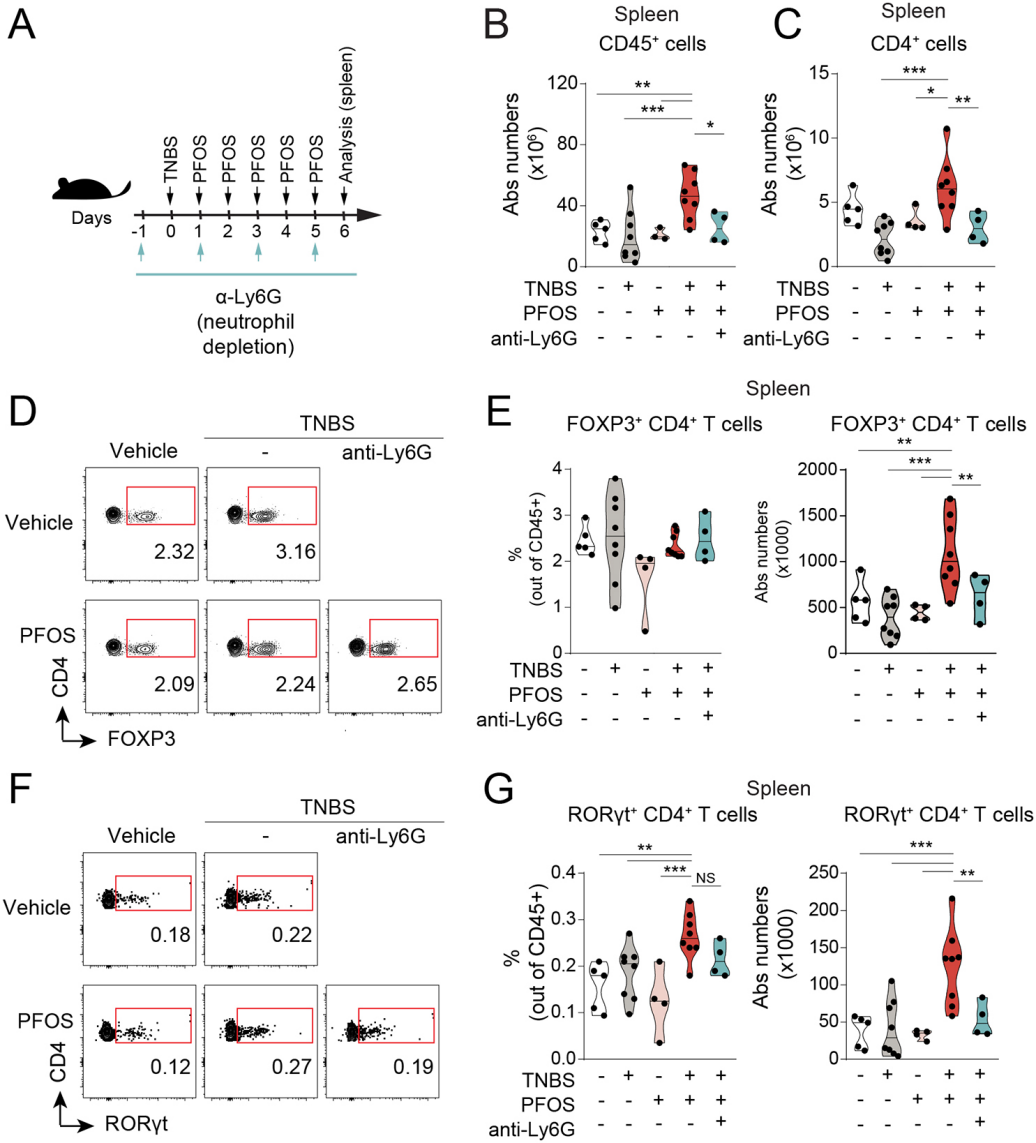
**Effects on T-cell homeostasis upon PFOS exposure are neutrophil dependent in colitic mice.** (A) Experimental outline. Mice received a single dose of TNBS (2.5%, in 50% ethanol, intrarectally administered), five doses of PFOS (10 mg/kg/total dose, orally gavaged) and 500 µg per dose of anti-Ly6G monoclonal antibody every other day. (B,C) Violin plots showing the absolute numbers of CD45^+^ (B) and CD4^+^ (C) T cells in spleen analyzed by flow cytometry. *n*=3-8, four experiments. (D-G) Flow cytometry analysis of FOXP3^+^ (D,E) and RORγt^+^ (F,G) CD4^+^ T cells in the spleen, following neutrophil depletion. Violin plots represent the absolute numbers and frequencies of these populations out of CD45^+^ cells. *n*=3-8, four experiments. The black line represents the median. NS, not significant; *P<0.05, **P<0.01, ***P<0.001. One-way ANOVA with Fisher's LSD test.

Previous studies have described that low doses of PFOS have minor effects on the composition of the immune compartment in the spleen and thymus in steady-state conditions (Qazi et al., 2009a,b), but how this is affected during colitis has not been addressed. We next asked whether the increase in peripheral PFOS levels during intestinal inflammation (Fig. 4A) might have an impact on systemic immune cell homeostasis. Analysis of the spleen showed that although PFOS alone did not alter the number of immune cells (CD45^+^ cells), these were significantly higher in PFOS-exposed mice undergoing intestinal inflammation (Fig. 5A,B), in particular CD4^+^ T cells (Fig. 5C). Because we showed that neutrophil numbers were increased in the intestine of PFOS-exposed mice during intestinal inflammation, we then asked whether neutrophil depletion might restore the systemic CD4^+^ T-cell levels in these mice. In agreement, we found that splenic CD4^+^ T cells were reduced in mice in which neutrophils had been depleted, reaching the same levels as those of non-PFOS-treated mice (Fig. 5C). Interestingly, we observed an increase in CD4^+^ T-cell, but not total CD45^+^ cell, numbers in the draining mesenteric lymph nodes (MLNs) from PFOS-exposed mice compared to those undergoing intestinal inflammation, which was neutrophil dependent (Fig. S7A,B).

We next assessed whether any specific T-helper effector subset was affected by the presence of PFOS. FOXP3^+^ CD4^+^ T cells were increased in numbers but not frequencies, while RORγt^+^ (also known as RORC^+^) CD4^+^ T cells were increased in frequencies and numbers, in colitic mice exposed to PFOS while undergoing colitis (Fig. 5D-G). However, we did not observe any differences in frequencies or numbers of T-cell subsets in the MLNs of PFOS-exposed mice that had undergone colitis (Fig. S7C,D). Altogether, our data suggest that PFOS exposure expands overall systemic CD4^+^ T-cell responses, with an increase in FOXP3^+^ and RORγt^+^ CD4^+^ T-cell numbers in the spleen.

**Fig. 6.**
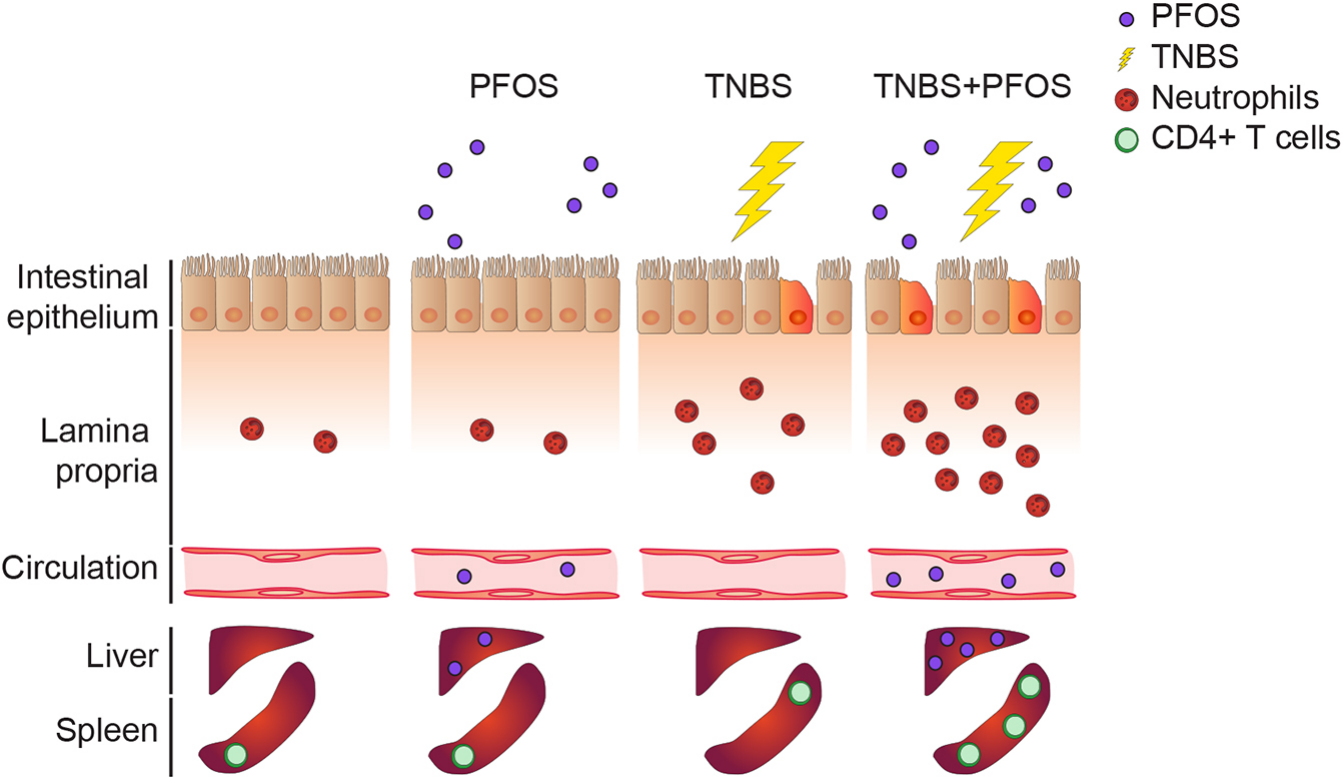
**PFOS exposure during intestinal inflammation enhances neutrophil-mediated damage and leads to CD4^+^ T-cell expansion in the periphery.** Scheme showing the proposed model in which PFOS exposure during ongoing intestinal inflammation leads to increased neutrophil recruitment, impairs epithelial barrier function and results in increased PFOS bioavailability and CD4^+^ T-cell numbers systemically.

Finally, we examined whether these changes in the composition of the T-cell effector subsets were neutrophil dependent. Indeed, we observed fewer RORγt^+^ CD4^+^ T cells in mice subjected to neutrophil depletion, as well as a reduction in absolute numbers of both FOXP3^+^ and RORγt^+^ CD4^+^ T cells (Fig. 5D-G), indicating that expansion of splenic CD4^+^ T cells as a result of PFOS exposure in colitic mice is neutrophil dependent. Altogether, we propose a model (Fig. 6) in which oral administration of PFOS during an ongoing intestinal inflammation results in disruption of barrier integrity and enhanced neutrophil recruitment, with subsequent increase in translocation of PFOS into the circulation and expansion of systemic CD4^+^ T cells.

## DISCUSSION

Identifying environmental factors that might lead to intestinal disorders is one of the main challenges in the IBD field (Ho et al., 2019). This study serves as a proof of principle for the use of zebrafish as a screening platform to examine the role of environmental pollutants in the modulation of intestinal inflammation in an intact organism *in vivo*, with subsequent validation and characterization of positive hits in mice.

PFASs are highly persistent and mobile man-made chemical pollutants that are commonly used in consumer products (Renner, 2001) and have been detected in wildlife and drinking water (Blum et al., 2015; Li et al., 2018). Although uptake through food and drinking water remains the most important route of exposure, it is still not completely understood how these compounds affect intestinal homeostasis. In this study, we assessed the effect of PFOS in zebrafish and mouse models of chemically induced intestinal inflammation, and found that it exacerbates neutrophil recruitment to the intestine while also increasing epithelial permeability, leading to a systemic expansion of CD4^+^ T cells in a neutrophil-dependent manner. Importantly, the combined exposure of PFOS during colitis increased the uptake of PFOS itself, resulting in higher internal doses in serum and target organs such as the liver. Considering the large number of adverse effects associated with PFAS exposure, this raises the question whether individuals with ongoing intestinal inflammation may be at higher risk of PFAS toxicity.

PFAS exposure has been associated with ulcerative colitis in humans (Steenland et al., 2013). However, the mechanisms underlying the correlation between PFASs and colitis are still unclear. Mounting health concerns have led to the replacement of longer-chain PFASs with their shorter-chain counterparts deemed less harmful, but experimental evidence of their immunotoxicity remains incomplete. Here, we tested both a longer-chain-length PFAS (PFOS; C8) and a shorter-chain-length compound with the same functional group (PFHxS; C6) in zebrafish, and found that PFOS exacerbated inflammation, in terms of expression of inflammatory cytokines, while PFHxS did not. This is in agreement with our previous report showing that shorter-chain-length PFASs induce less toxicity in zebrafish larvae than their longer-chain counterparts upon exposure to the same concentration, which was associated with different internal concentrations in larvae (Vogs et al., 2019). However, although less bioaccumulative (Conder et al., 2008), it is still unclear if prolonged exposure to short-chain PFASs, or an exposure that would lead to the same internal concentration, could also bring detrimental effects, and this thus requires further investigation. Finally, although we did not find major changes in cytokine expression following PFOS exposure at this concentration, we cannot exclude that doses above the LOEL, or in combination with additional environmental factors, might significantly affect cytokine production.

The effect of PFOS on innate immune responses has been partially studied, as PFOS has been shown to promote antimicrobial defense at early stages of *Citrobacter rodentium* infection in the large intestine by enhancing IL-22 production by innate lymphoid cells, but to cause persistent inflammation and limit bacterial clearance at later stages (Suo et al., 2017). However, its effect on the immune response elicited by myeloid cells in the intestinal mucosa and on the intestinal epithelial barrier is largely unknown. Specifically, neutrophils are one of the first cell types to arrive to a site of injury or infection (Amulic et al., 2012) and are a major sign of intestinal inflammation in humans (Muthas et al., 2017). Here, we observed increased recruitment of neutrophils to the digestive tract of zebrafish and mice exposed to PFOS while undergoing intestinal inflammation. This was also associated with increased histopathological score and body weight loss in mice, indicating that the co-exposure led to a worsened colitis. In addition, colitic mice showed an increase in intestinal permeability when exposed to PFOS, which was not observed in non-colitic mice exposed to the pollutant. This also correlated with increased levels of PFOS systemically in colitic mice. These findings suggest that pollutant exposure can affect the function of the intestinal barrier and result in further PFOS bioaccumulation. Hence, with these results, we could propose a model in which increased neutrophil recruitment to the intestinal tract upon exposure to PFOS in the context of intestinal inflammation results in prolonged inflammation and delayed restitution of barrier integrity.

Considering the increased systemic PFOS uptake and ongoing colonic inflammation, we wondered whether this could have an impact on the immune cell compartment in extra-intestinal organs. Yet, we did not find any major changes in the immune compartment of the spleen in mice treated with PFOS alone. Nevertheless, colitic mice exposed to PFOS showed an expansion of total CD4^+^ T cells. These results could be partly due to inflammatory cues stemming from the intestine in colitic mice together with the increased PFOS levels in the periphery. Interestingly, we observed an increase in total CD4^+^ T cells in the MLNs in PFOS-treated mice, suggesting that PFOS exposure might affect local T-cell responses in the intestine.

Neutrophil migration and removal from sites of injury must be tightly controlled, because failure in these mechanisms can lead to tissue damage found in chronic inflammatory diseases (Amulic et al., 2012). Thus, we hypothesize that the exacerbated neutrophil recruitment to the intestine upon PFOS exposure during colitis might affect biological processes involved in the resolution of inflammation and intestinal barrier recovery. The reduced FITC-Dextran levels observed in the TNBS+PFOS group after neutrophil reduction suggest that the increased intestinal permeability found in the TNBS+PFOS group is due to the exacerbated neutrophil infiltration. Furthermore, the reversion of CD4^+^ T-cell expansion in the spleen after neutrophil depletion in the TNBS+PFOS group indicates that neutrophils not only affect intestinal homeostasis, but also promote an extra-intestinal T-cell response in response to PFOS during colitis. Overall, this highlights a key finding on how exacerbation of intestinal inflammation due to exposure to a widely distributed pollutant can also impact extra-intestinal organs. However, whether this increased systemic inflammatory state would predispose to extra-intestinal conditions is beyond the scope of this study and requires further analysis. Moreover, considering the global increase in the prevalence of inflammatory diseases and the large number of individuals estimated to be exposed to PFAS amounts exceeding the tolerable weekly intake, further studies to delineate the risks of PFASs on immune function and the underlying mechanisms are warranted.

Considering that mice infected with *C. rodentium* and exposed to PFOS showed differences at late, but not early, stages of infection (Suo et al., 2017), we wondered whether PFOS exposure would affect the composition of the intestinal microbiota in mice undergoing TNBS-induced colitis. Although we found similar levels of different microbial taxa in colitic mice exposed to PFOS or not at day 3 after TNBS administration, we cannot exclude that those changes in the microbial composition could become apparent at later stages, as previously reported (Suo et al., 2017). Moreover, our analysis of the intestinal composition and structure did not show evident differences between groups, which is in line with a previous study characterizing the TNBS inflammation model in zebrafish (Oehlers et al., 2011).

In this study, we used zebrafish larvae as an *in vivo* model to analyze the effects of environmental pollutants on immune responses. Although innate immune cells and T cells are present in the intestine at this developmental stage (Coronado et al., 2019), it is unclear if the latter respond to antigen recognition, especially because the thymus is not fully mature at this time point (Lam et al., 2002). Furthermore, we visualized increased neutrophils and macrophages in the intestine of zebrafish larvae, but these did not correlate with transcript levels of the markers used, which can be due to the low transcript levels compared with the accumulation of fluorescent reporter protein in these cells. Despite the limitation in studying the contribution of the adaptive immune response, our model provided significant insights in the innate counterpart, which might play a dominant role in triggering intestinal inflammation in IBD patients.

In summary, using zebrafish and mice, we here demonstrated that exposure to an environmental pollutant exacerbates intestinal inflammation, which might result in systemic effects. These findings can be further investigated to examine the consequence of such changes in extra-intestinal autoimmune diseases, particularly those in which T-helper cells have a predominant role, such as experimental autoimmune encephalomyelitis. We believe that the integration of the zebrafish model with existing models will contribute to advancing towards the development of therapeutic intervention and treatment strategies.

## MATERIALS AND METHODS

### Animal husbandry

C57Bl/6 mice were purchased from Charles River Laboratories or Taconic. All mice were sex matched and 6-8 weeks old. Colitis was induced by intrarectal administration of 100 µl TNBS (1-2.5%, depending on the experiment, in 50% ethanol; Sigma-Aldrich, St Louis, MO, USA) (Wirtz et al., 2007) using a flexible oral gavage needle inserted in the colon ∼4 cm proximal to the anus. During the administration, mice were anesthetized with isofluorane. Mice were held vertically for 1 min following the procedure and monitored until they recovered. Control mice were treated identically but were instilled with water. PFOS (Sigma-Aldrich) was dissolved in dimethyl sulfoxide and stored at −20°C. For *in vivo* treatments, PFOS was dissolved in water containing 0.5% Tween 20, and mice were gavaged with 200 µl every day (5 mg/kg/total accumulated dose for analysis at day 1 following TNBS treatment, and 10 mg/kg/total dose for mice analyzed at day 3 and 6). Animals were kept under specific pathogen-free conditions and handled according to protocols approved by the Stockholm Regional Ethics Committee (Dnr 3227-2017). All experiments were performed following the national and institutional guidelines and regulations.

Zebrafish (*Danio rerio*) were obtained from natural spawnings between Tg(*lysC:DsRed2*, here referred to as *lyz:DsRed2*) (Hall et al., 2007), TgBAC(*cldn15la:GFP*, here referred to as *cldn15la:GFP*) (Alvers et al., 2014) and wild type (AB strain, provided by the Karolinska Institutet Zebrafish Core Facility, Stockholm, Sweden) under the protocols approved by the Stockholm Regional Ethics Committee (Dnr 5.2.18-12891/15, N220/15 and 14049-2019). Larvae were raised at 28°C in E3 water. The medium for *lyz:DsRed2* larvae was also supplemented with 30 mg/l phenylthiourea to avoid pigmentation. Larvae were exposed to DSS (0.5%, Affymetrics), TNBS (50 µg/ml or 70 µg/ml) and PFOS, PFOA or PFHxS (200 nM; Sigma-Aldrich), diluted to working concentrations in E3 water, from 72 hpf to 120 hpf. Exposures were performed in 1 ml medium/well in Falcon^®^ 24-well polystyrene plates, 10 larvae/well. The exposure medium was renewed at 96 hpf.

### FITC-Dextran permeability assay

Evaluation of the intestinal permeability by FITC-Dextran was performed as previously described (Czarnewski et al., 2019). Mice were gavaged with 10 mg/ml FITC-Dextran (Sigma-Aldrich) at day 3 after the TNBS administration. After 4 h, mice were sacrificed, and the blood was collected. Serum was diluted 1:10 in PBS and added to a 96-well plate for fluorescent-based assays (Invitrogen). Fluorescence was quantified on a fluorescent plate reader with a 535/587-nm excitation/emission filter. The concentration of FITC-Dextran was calculated by interpolation to an 8-point dilution standard curve.

### Neutrophil depletion

To deplete neutrophils, mice were injected intraperitoneally every other day with 500 µg per dose of anti-Ly6G monoclonal antibody (clone 1A8, BioXCell) or isotype control (clone 2A3, BioXCell), as indicated in each figure, as described in Jamieson et al. (2012).

### Isolation of leukocytes and flow cytometry

Colon lamina propria cells were isolated as previously described (Parigi et al., 2018). Cells from the spleen were isolated by smashing the tissues through a 70 µm cell strainer. Single-cell suspensions were incubated at 4°C for 15 min with Fc blocking antibody (1:1000; anti-CD16/32 antibody, eBioscience), followed by staining with fluorochrome-conjugated antibodies at 4°C for 15 min. For staining of myeloid cells in the colon lamina propria, the following cocktail was used: anti-CD45.2 (1:200; 104, eBioscience), anti-CD11b (1:200; M1/70, eBioscience), anti-MHCII (1:200; M5/114.15.2, eBioscience), anti-Ly6C (1:200; HK1.4, eBioscience), anti-CD11c (1:200; N418, BioLegend), anti-CD64 (1:200; X54-5/7.1, BioLegend), anti-Ly6G (1:200; 1A8, BD Biosciences). Fixable Viability Dye eFluor^®^ 506 (1:1000; eBioscience) was used to exclude dead cells. For staining of T cells in the spleen, we used the following cocktail: anti-CD45.2 (1:200; 104, eBioscience), anti-CD3 (1:200; 17A2, BioLegend), anti-CD4 (1:200; GK1.5, BioLegend), anti-FOXP3 (1:200; FJK-16S, eBioscience), anti-Gata3 (1:200; TWAJ, eBioscience), anti-RORγt (1:200; Q31-378, BD Biosciences), anti-T-bet (1:200; 4B10, BioLegend), Fixable Viability Dye eFluor™ 780 (1:1000; eBioscience). The dilution of antibodies used is the lowest that provides sufficient separation between positive and negative populations in our hands. Most of these antibodies have also been used in previous studies in our laboratory (Czarnewski et al., 2019; Parigi et al., 2018). Acquisition was performed using FACS Canto II or LSR Fortessa flow cytometers (BD Biosciences) and analyzed using FlowJo software (Tree Star, Ashland, OR, USA).

### Zebrafish live imaging and image analysis

Following washing in E3 medium, 5 days post-fertilization (dpf) *lyz:DsRed2* and *mpeg1:mCherry-F* larvae were anesthetized with 0.016% Tricaine (MS-222, Sigma-Aldrich) and positioned in Petri dishes for imaging. Epi-fluorescence microscopy was performed using a SMZ25 Research Stereo Microscope (Nikon) with NIS Elements Basic Research Imaging Software, ver. 4.30 (Nikon). Images were cropped to contain the region of interest, and the number of DsRed2^+^ cells and mCherry^+^ cells was analyzed automatically using a custom pipeline (available upon reasonable request) with the open-source software CellProfiler ver. 2.2.0 (Broad Institute, Cambridge, MA, USA; www.cellprofiler.org). The automated quantification was verified by manual screening of CellProfiler-processed images to identify cell counts for image areas with low signal-to-noise ratio (i.e. few cells), which were excluded. Confocal images were taken with a Zeiss LSM800 microscope (Zeiss, Germany). The number of DsRed2^+^ cells in the intestine (identified as the GFP^+^ area) was manually counted. The intensity of the GFP^+^ area is reported as mean intensity (the sum of pixel intensities/number of pixels) and was analyzed automatically using a custom pipeline with the open-source software CellProfiler ver. 2.2.0. Light sheet images were acquired using a Light Sheet Z.1 (Zeiss) with a 5× air-detection objective (EC Plan-Neofluar 5×/0.16 NA) and dual-side 5× illumination objectives (LSFM, 0.1 NA).

### Alcian Blue staining of zebrafish larvae

At 5 dpf, and after 48 h of treatment, zebrafish larvae were rinsed three times with E3 medium and fixed in 4% paraformaldehyde in PBS at 4°C overnight. Alcian Blue staining was performed as described previously (Oehlers et al., 2013), with an additional step of depigmentation with H_2_O_2_ 1.5%/KOH 0.5% in water after staining. Larvae were mounted in a lateral position using l% low-gelling point agarose (Sigma-Aldrich), and RGB images were acquired using a Nikon SMZ25 stereoscope equipped with a DS-Fi3 camera. Images were cropped to keep the mid-intestine section. Automatic analysis of Alcian Blue-stained area in the intestine was performed in Fiji software using the ‘Colour Deconvolution 1.7’ option, and selecting the ‘Alcian blue & H’ vector to identify the Alcian Blue-stained area.

### Paraffin inclusion and H&E staining of zebrafish larvae

Five days post-fertilization zebrafish larvae were rinsed three times with E3 medium and fixed in 4% paraformaldehyde in PBS at 4°C overnight. The larvae were washed three times in PBS, for 20 min each, then dehydrated in an ascending ethanol series (25-100%) for 40 min each and immersed twice in xylene for 40 min. Larvae were embedded in paraffin, and 5 μm sections were cut (Microm HM355S, Thermo Fisher Scientific, Germany) for staining. Deparaffination was performed by incubating the sections at 60°C for 30 min followed by two clearing steps in xylol of 10 min each. Sections were progressively rehydrated and stained with Harris Hematoxylin (Sigma-Aldrich) and Eosin-Y (Merck, Germany) following a standardized protocol for zebrafish tissues (https://zebrafish.org/wiki/health/disease_manual/recipes_and_protocols; Zebrafish International Resource Center, USA). Slides were progressively dehydrated in propanol and xylene prior to mounting in DPX Mountant for histology (Merck, Germany). Images were taken using a widefield microscope (AxioImager M2, Zeiss, Germany) using the software Cell^M (Olympus, Germany).

### Real-time qPCR

Pools of ten zebrafish larvae, dissected intestines or carcasses were collected in TRIzol LS reagent (Invitrogen) and homogenized by passing ten times through a 23-gauge needle and ten times through a 27-gauge needle. For dissection of digestive tracts, larvae were euthanized and intestines were dissected as described (Bates et al., 2006). Total RNA was isolated according to the manufacturer's protocol. Reverse transcription was performed using an iScript cDNA Synthesis kit (Bio-Rad), which contains a blend of oligo(dT) and random hexamer primers.

DNA was extracted from colonic stools using a QIAamp DNA Stool Mini Kit (Qiagen), following the manufacturer's protocol. iQ SYBR Green Supermix (Bio-Rad) was used for qPCR. mRNA levels were determined using a CFX 384™ Touch Real-Time PCR System (Bio-Rad). Reaction conditions consisted of 40 cycles of PCR with 58°C annealing temperature for zebrafish primers and 60°C for bacterial primers. Relative quantities of mRNA were calculated using the ΔΔCt method, by normalization to *eef1a1l1* for zebrafish genes and to universal 16S levels for bacterial abundance (Jijon et al., 2018; Yang et al., 2015), and expressed as log2-transformed fold change relative to untreated control. Primers used to analyze zebrafish genes and bacterial abundance are listed in Tables S1 and S2, respectively.

### Histological scoring

Sections 0.5 cm from the distal colon were collected, rinsed in PBS and fixed in 4% formaldehyde solution for 24 h, prior to paraffin embedding. Sectioning and H&E staining were performed as indicated in Czarnewski et al. (2019). Scoring was performed by a pathologist at the Unit for Morphological Phenotype Analysis (FENO) at the Karolinska Institute, in a blind manner and following the system described for this model (Wirtz et al., 2017).

### Chemical analysis

#### Sample preparation

Liver tissues were sectioned, and ∼100 mg from respective samples was transferred to 2 ml centrifuge tubes. Internal standard (IS) solution in 70% acetonitrile was added at a ratio of three parts IS solution to one part tissue, i.e. 300 µl IS solution per 100 mg tissue. The tissues were homogenized in the IS solution using a TissueLyser (Qiagen) with a 0.5 mm stainless steel bead for 1 min at 15 Hz, followed by vigorous shaking at room temperature for 30 min, and finally 10 min centrifugation at 1600 ***g***. Tissue extracts were diluted 1:100 in 75% acetonitrile/water, and 0.150 ml of mixture was transferred to a glass insert (Teknolab Sorbent, Kungsbacka, Sweden) of a 96-well Rittner plate (Teknolab Sorbent). Serum samples were also diluted 1:100 in 75% acetonitrile/water, and 0.050 ml of serum dilution was added together with 0.150 ml of IS solution into a glass insert (Teknolab). The plates were centrifuged for 30 min at 3000 ***g*** prior to analysis.

#### Liquid chromatography–tandem mass spectrometry (LC-MS/MS) instrumentation and conditions

Four microliters of diluted extracts or serum were injected onto the LC-MS/MS. A 4 µm C18 column [2.1 mm inner diameter (i.d.)×50 mm; Genesis Lightning] was used before the injector to reduce the interference of contaminants during the mobile phase. A 1.7 µm C18 column (2.1 mm i.d.×100 mm; Fortis Technologies) was used for analysis, and the mobile phases were water (A) and acetonitrile (B), with 0.1% formic acid. The samples were analyzed on a Shimadzu UFLC system (Shimadzu Corporation, Kyoto, Japan) coupled to a QTRAP5500 (triple quadrupole linear ion trap mass spectrometer) equipped with a TurboIon Spray source (AB Sciex, Framingham, MA, USA), according to Xu et al. (2020).

Ten microliters of diluted extracts or serum were injected onto the LC-MS/MS. A 1.7 µm C18 column (2.1 mm i.d.×100 mm; Fortis Technologies) was used before the injector to reduce the interference of contaminants during the mobile phase. A 4 µm C18 column (2.1 mm i.d.×50 mm; Genesis Lightning) was used for analysis, and the mobile phases were water and acetonitrile with 0.1% formic acid. The samples were analyzed on a Shimadzu UFLC system (Shimadzu Corporation) coupled to a QTRAP5500 (triple quadrupole linear ion trap mass spectrometer) equipped with a TurboIon Spray source (AB Sciex). All samples were analyzed in technical duplicates, all runs included at least ten blank samples, and background contamination was subtracted from all values. Excellent linearity was seen for the calibration standards ranging from 0 to 1000 ng/ml in methanol.

### Statistical analysis

Plots and statistical analysis were performed using Prism 8 software (GraphPad, San Diego, CA, USA). All data sets were analyzed by one-way ANOVA followed by Fisher's least significant difference (LSD) (α=0.05) in all plots except those in which only two groups are presented, which were analyzed with an unpaired Student's *t*-test. Statistically significant outliers were identified using the ROUT method. In violin plots, the black line represents the median. Error bars in dot plots represent mean±s.d.

## Supplementary Material

Supplementary information
