# Supplementary material for: Perfluorooctanesulfonic acid modulates barrier function and systemic T-cell homeostasis during intestinal inflammation
Source: Dis Model Mech. 2021 Dec 23;14(12):dmm049104. doi: 10.1242/dmm.049104 (PMC8713990; doi:10.1242/dmm.049104)
Supplement: Supplementary information [file dmm-14-049104-s1.pdf]

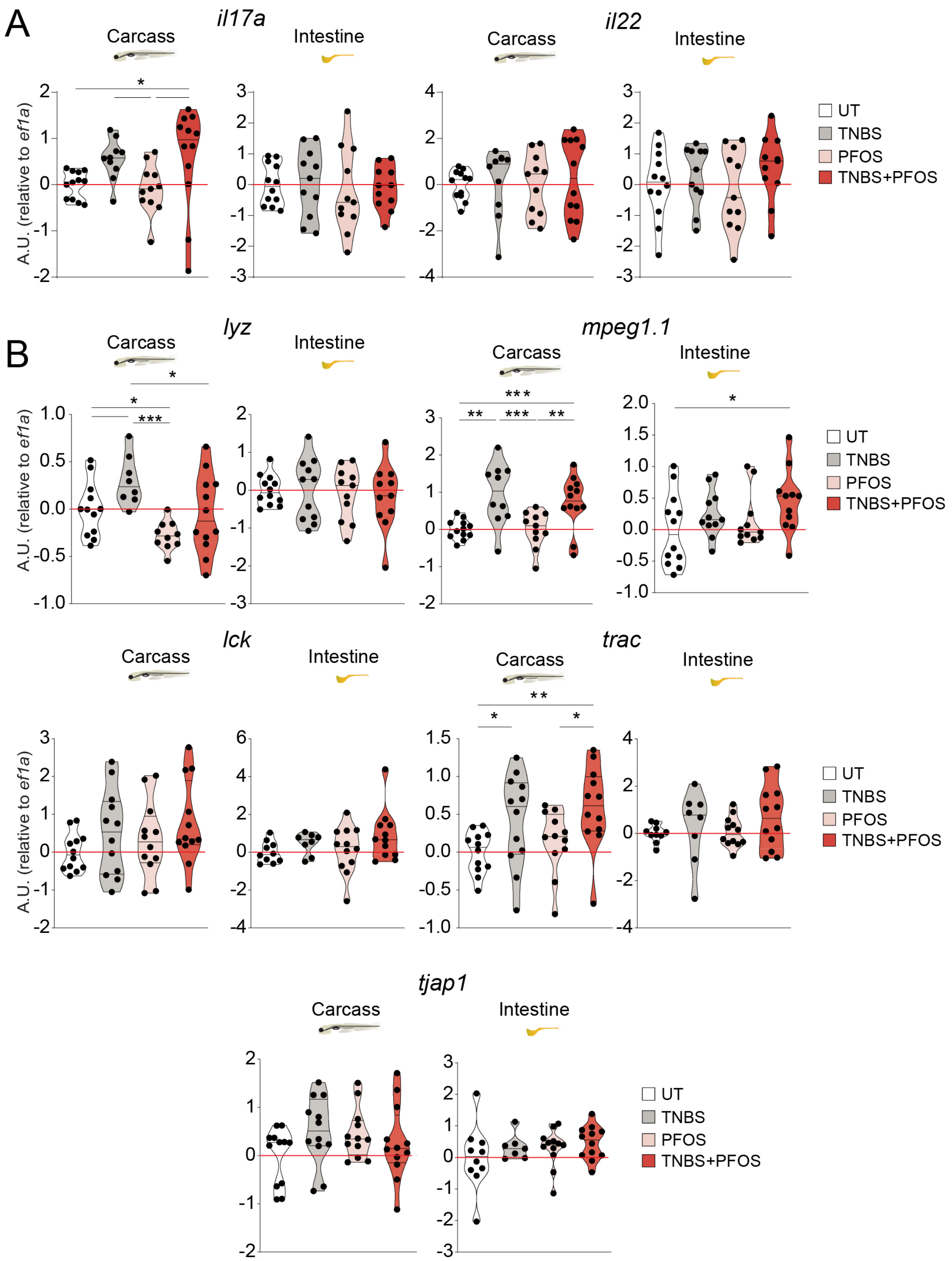

Fig. S1. PFOS does not alter *il17a* or *il22* expression in zebrafish larvae during TNBS-induced inflammation. (**A-B**) Violin plots showing relative expression of *il17a, il22, lyz, mpeg1.1, lck, trac,* and *tjap1* analyzed by qPCR in dissected intestines or carcasses at 120 hpf following exposure to TNBS (70 µg/mL) and PFOS (200 nM). n = 11-12, 4 experiments. Data show transcript levels as A. U. respect to *eef1l1a1*. The black line represents the median. * $p < 0.05$, ** $p < 0.01$, *** $p < 0.001$. One-way ANOVA with Fisher's Least Significant Difference (LSD).

Disease Models & Mechanisms • Supplementary information

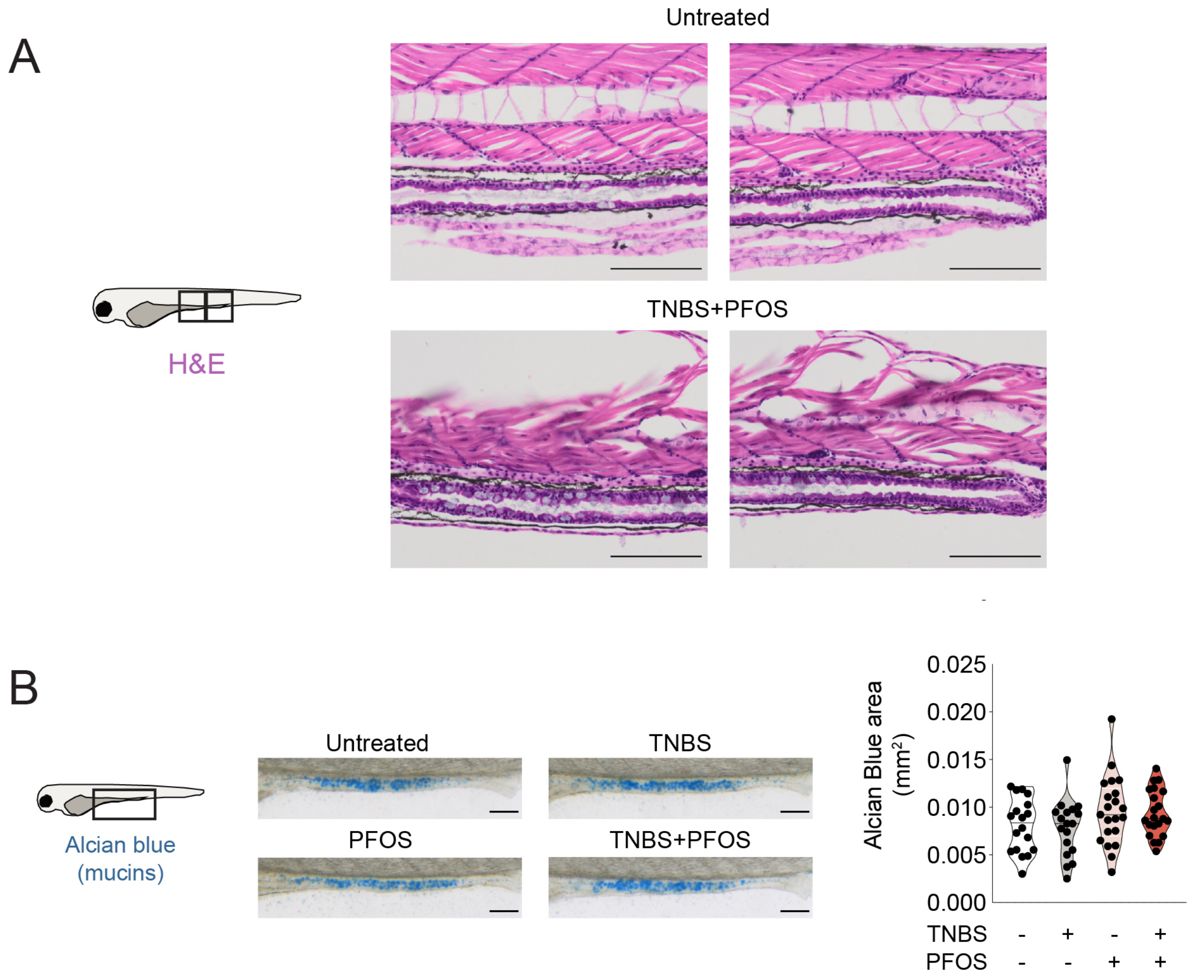

Fig. S2. PFOS exposure does not alter intestinal epithelial architecture or mucin production. (A) Representative images of hematoxylin & eosin (H&E) stained 5 days post-fertilization larvae following 48 hours exposure to TNBS (70 µg/mL) and PFOS (200 nM). **(B)** Whole-mount alcian blue staining of zebrafish embryos treated with TNBS+PFOS. The graph represents the quantification of the alcian blue positive area per intestinal area, as described in the cartoon. *n* = 18-21, 1 experiment. Each data point represents one 120 h post fertilization zebrafish larvae. The black line represents the median. One-way ANOVA with Fisher's Least Significant Difference (LSD).

Disease Models & Mechanisms • Supplementary information

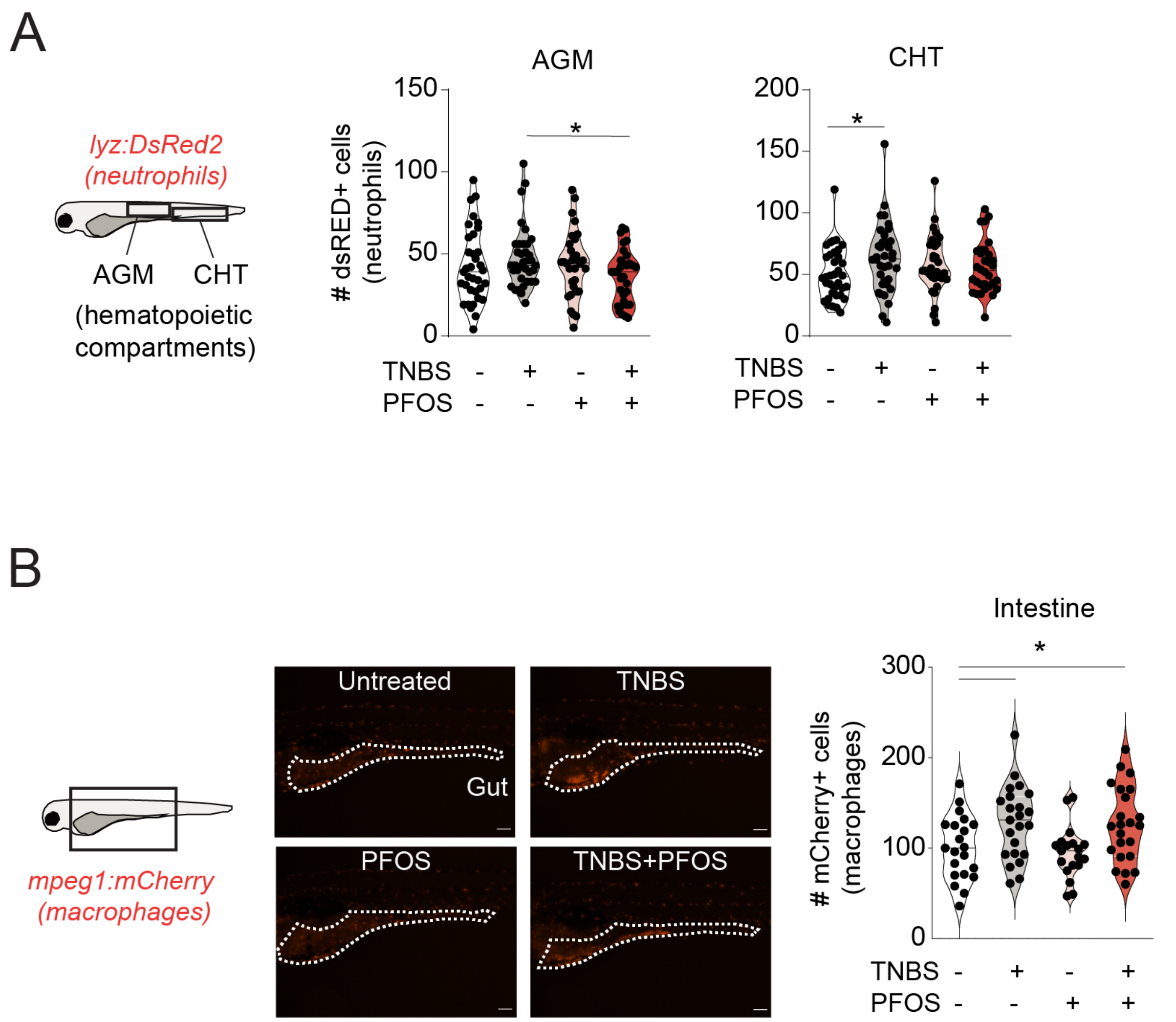

Fig. S3. PFOS does not alter neutrophils numbers at hematopoietic sites nor macrophages intestinal numbers during TNBS-induced inflammation in zebrafish larvae. (A) Quantification of DsRed2-positive cells (neutrophils) in the hematopoietic regions (AGM; aorta-gonad-mesonephros, and CHT; caudal hematopoietic tissue) as described in the cartoon. *n* = 32-35, 5 experiments. Each data point represents one 120 h post fertilization zebrafish larvae. (**B)** Quantification of mCherry-positive cells (macrophages) in the gastrointestinal tract of 120 h post fertilization *mpeg1:mCherry-F* larvae. Representative images are 7x magnification. The dash line demarks the gastrointestinal tract area. n = 19-23, 2 experiments. Each data point represents one 120 h post fertilization zebrafish larvae. The black line represents the median. * $p < 0.05$. One-way ANOVA with Fisher's Least Significant Difference (LSD).

Disease Models & Mechanisms • Supplementary information

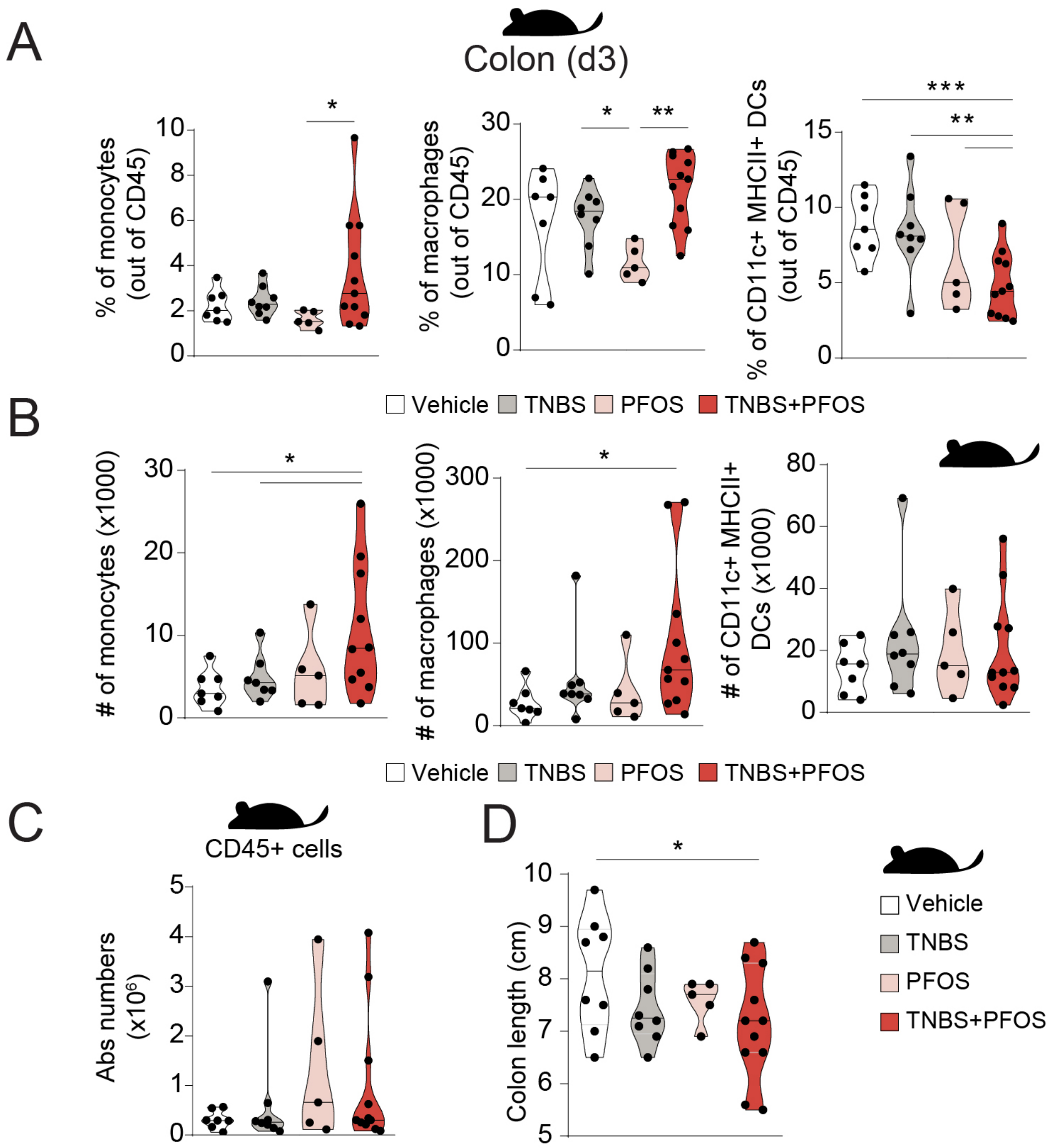

Fig. S4. Myeloid cell characterization of PFOS exacerbated colitis in the colon. **(A-C)** Violin plots showing frequencies out of CD45^{+} cells and absolute numbers of myeloid cells in the colon lamina propria, analyzed by flow cytometry. n = 5-11, 3 experiments. **(D)** Violin plots showing colon length of mice treated with PFOS and TNBS as described in Figure **3D**. The black line represents the median. * $p < 0.05$, ** $p < 0.01$, *** $p < 0.001$. One-way ANOVA with Fisher's Least Significant Difference (LSD) test.

Disease Models & Mechanisms • Supplementary information

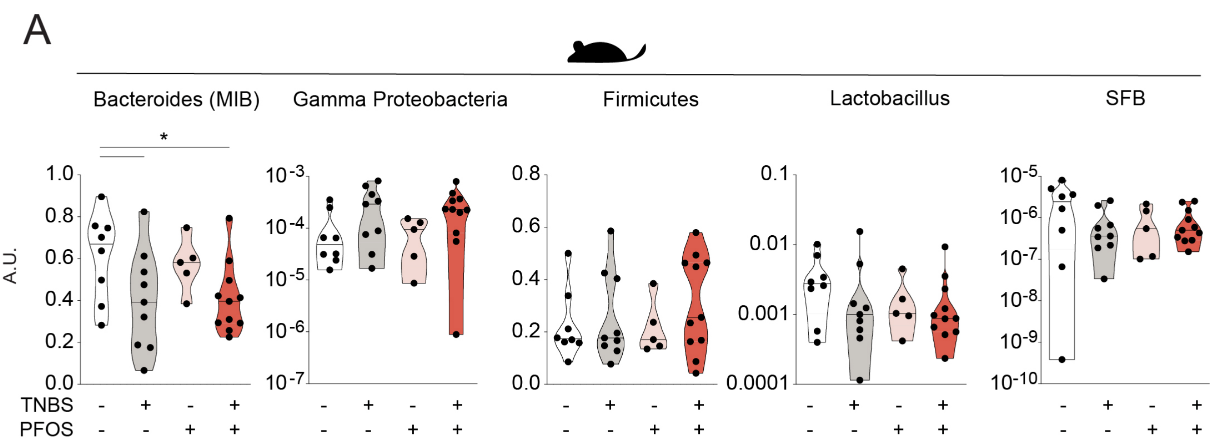

Fig. S5. PFOS exposure results in reduced abundance of Bacteroides. (**A**) Violin plots showing relative abundance of Bacteroides (Mouse Intestinal Bacteroides, MIB), Gamma Proteobacteria, Firmicutes, *Lactobacillus*, and segmented filamentous bacteria (SFB) relative to universal 16S levels analyzed by qPCR. Bacterial DNA was isolated from stool obtained from the colon. n = 5-11, 3 experiments. The black line represents the median. * $p < 0.05$. One-way ANOVA with Fisher's Least Significant Difference (LSD) test.

Disease Models & Mechanisms • Supplementary information

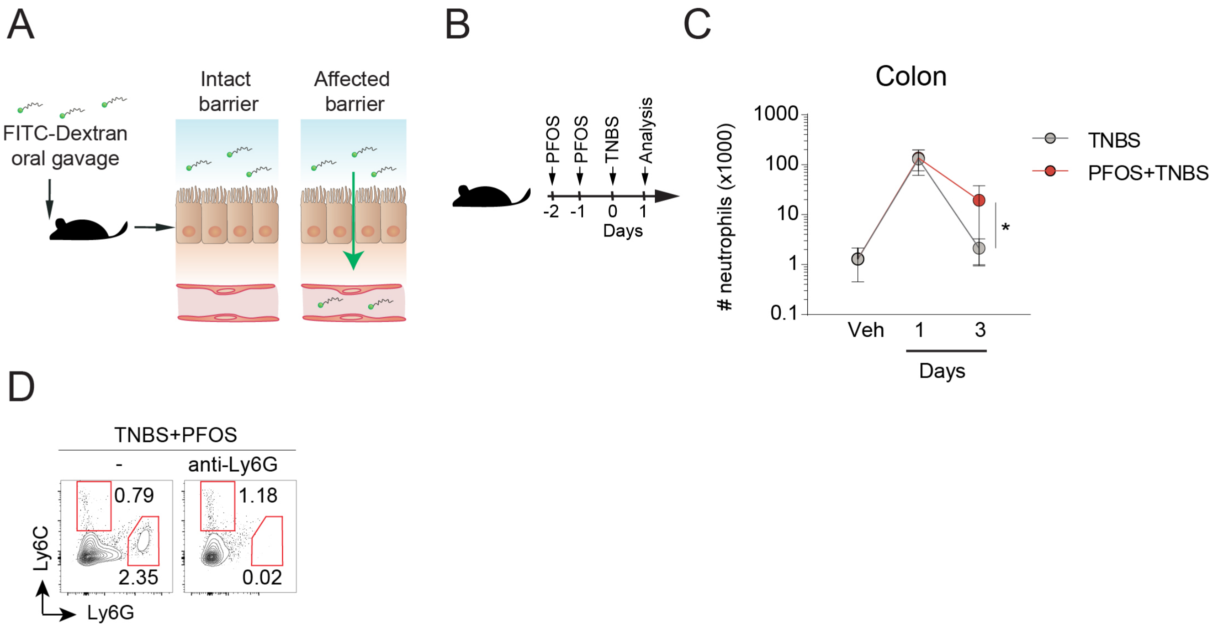

Fig. S6. PFOS does not alter neutrophil numbers or frequencies in the colon 1 day after TNBS-induced colitis and these are efficiently depleted with anti-Ly6G antibody treatment. (A) Experimental scheme of the FITC-Dextran permeability assay. **(B)** Experimental outline. (**C**) Absolute numbers of neutrophils isolated from the colon lamina propria at day 1 and 3 following TNBS administration. n = 7-9, 3 experiments for day 1. Data for day 3 corresponds to the data shown in Figure **3F**, n = 5-11, 3 experiments. Veh; vehicle group from the experiment performed at day 1, thus the same values are used for both TNBS and TNBS+PFOS. (**D**) Representative dot plots of flow cytometry analysis of neutrophils isolated from the colon lamina propria at day 3 following neutrophil depletion in TNBS+PFOS treated mice. Neutrophils are gated as Ly6C^{int} and Ly6G^{+}. Monocytes are gated as Ly6C^{+} and Ly6G^{-}. * $p < 0.05$. One-way ANOVA with Fisher's Least Significant Difference (LSD) test.

Disease Models & Mechanisms • Supplementary information

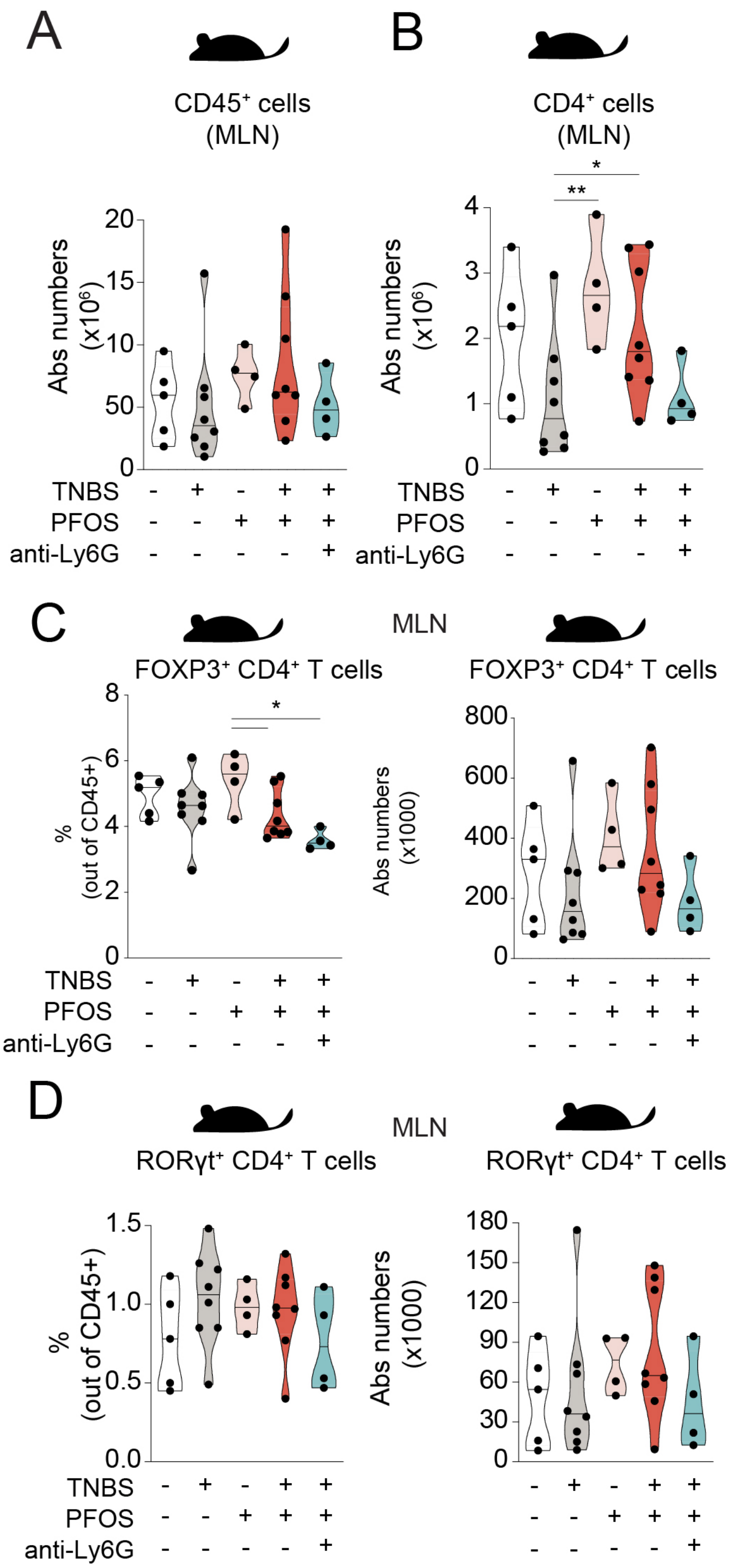

Fig. S7. PFOS exposure after TNBS-induced colitis results in increased CD4$^+$ T cells in the MLN. (**A-D**) Flow cytometry analysis of MLN FOXP3$^+$ and RORγt$^+$ CD4$^+$ T cells, following TNBS, PFOS and/or neutrophil depletion as in the scheme shown in Figure 5A. Violin plots represent the absolute numbers and frequencies of these populations out of CD45$^+$ cells. n = 4-8, 4 experiments. The black line represents the median. * $p < 0.05$, ** $p < 0.01$, *** $p < 0.001$. One-way ANOVA with Fisher's Least Significant Difference (LSD) test.

Disease Models & Mechanisms • Supplementary information

Table S1. List of primers used in this study for zebrafish genes

Target gene	ENSEMBL accession number	Forward sequence (5' -> 3')	Reverse sequence (5' -> 3')
il1b	ENSDARG00000098700	ATCAAACCCCAATCCACAGAGT	GGCACTGAAGACACCACGTT
tnfa	ENSDARG00000009511	GGAGAGTTGCCTTTACCGCT	TTGCCCTGGGTCTTATGGAG
eef1a1l1	ENSDARG00000020850	ACCTACCCTCCTCTTGGTCG	GGAACGGTGTGATTGAGGGAA
il17a/f3	ENSDARG00000041976	CGCCTTGGACATACACAACTT	AGTAAATGGGTTGGGACTCCA
il22	ENSDARG00000045673	CGATGACTGATACAGCACGA	TGTGCTCGTCTGATTCCAAG
cldn15la	ENSDARG00000016081	TGTTTGATCGGCTGGGTTCT	AGCCTGAATGTGAACTGGCAA
lyz	ENSDARG00000114098	AGCAGGTTTAAGACCCACCG	AGCAAGTCTGAACAGGCCAC
mpeg1.1	ENSDARG00000055290	ATGTCGTGGCTGGAACAGAA	ATGGTTACGGACTTGAACCC
lck	ENSDARG00000102525	GCCGAAGAAGATCTCGATGGT	TCCCCATGTTTACGTATTTTGTCG
trac	ENSDARG00000104132	CTGCGAGGAGACAGGCGTTT	GCCAGAAGATGCCCAGTGAC
tjap1	ENSDARG00000010681	CTCACCAATAAACTTGTGGACGC	TGTCGATAGCGTTCGTTCAGC

Table S2. List of primers used in this study to analyze bacterial abundance

Target group	Forward sequence (5' -> 3')	Reverse sequence (5' -> 3')
Universal 16S rRNA	ACTCCTACGGGAGGCAGCAGT	ATTACCGCGGCTGCTGGC
Bacteroides (MIB)	CCAGCAGCCGCGGTAATA	CGCATTCCGCATACTTCTC
Gamma Proteobacteria	GCTAACGCATTAAGTRYCCCG	GCCATGCRGCACCTGTCT
Firmicutes	GGAGYATGTGGTTTAATTCGAAGCA	AGCTGACGACAACCATGCAC
Lactobacillus	AGCAGTAGGGAATCTTCCA	CACCGCTACACATGGAG
Segmented filamentous bacteria (SFB)	GACGCTGAGGCATGAGAGCAT	GACGGCACGGATTGTTATTCA

Disease Models & Mechanisms • Supplementary information
